# Phosphorylations of Serines 21/9 in Glycogen Synthase Kinase 3α/β Are Not Required for Cell Lineage Commitment or WNT Signaling in the Normal Mouse Intestine

**DOI:** 10.1371/journal.pone.0156877

**Published:** 2016-06-10

**Authors:** Fiona Hey, Susan Giblett, Stephanie Forrest, Chelsea Herbert, Catrin Pritchard

**Affiliations:** 1 Department of Molecular Cell Biology, Henry Wellcome Building, University of Leicester, Leicester, United Kingdom; 2 Department of Cancer Studies, Robert Kilpatrick Clinical Sciences Building, University of Leicester, Leicester, United Kingdom; University of Kentucky, UNITED STATES

## Abstract

The WNT signalling pathway controls many developmental processes and plays a key role in maintenance of intestine renewal and homeostasis. Glycogen Synthase Kinase 3 (GSK3) is an important component of the WNT pathway and is involved in regulating β-catenin stability and expression of WNT target genes. The mechanisms underpinning GSK3 regulation in this context are not completely understood, with some evidence suggesting this occurs through inhibitory N-terminal serine phosphorylation in a similar way to GSK3 inactivation in insulin signaling. To investigate this in a physiologically relevant context, we have analysed the intestinal phenotype of GSK3 knockin mice in which N-terminal serines 21/9 of GSK3α/β have been mutated to non-phosphorylatable alanine residues. We show that these knockin mutations have very little effect on overall intestinal integrity, cell lineage commitment, β-catenin localization or WNT target gene expression although a small increase in apoptosis at villi tips is observed. Our results provide *in vivo* evidence that GSK3 is regulated through mechanisms independent of N-terminal serine phosphorylation in order for β-catenin to be stabilised.

## Introduction

The small intestine is comprised of the crypts of *Lieberkühn*, invaginations of the epithelium containing pluripotent stem cells, and of villi, finger-like protrusions containing differentiated cells. The intestinal epithelium renews every 3–5 days in adults and this is achieved through the activity of the crypts [1]. The intestinal stem cells (ISCs), which reside at crypt bottoms, are the driving force behind intestinal renewal [2]. Their proliferative progenitors, transit amplifying (TA) cells, undergo 4–5 divisions before terminally differentiating [3]. During this process, the TA cells migrate upwards towards the villus base and differentiate into absorptive or secretory cells, namely enterocytes, goblet cells and enteroendocrine cells. These differentiated cells continue to move upwards and, upon reaching the villus tip, they undergo apoptosis and are shed into the gut lumen. The Paneth cell represents a fourth cell type, also derived from the ISCs, but this cell type migrates in the opposite direction into the crypt base [4, 5].

At the crypt base is the ‘stem cell zone’ created by ISCs or crypt base columnar (CBC) cells interspersed between the Paneth cells [6]. These stem cells can be identified by their expression of key marker genes including *Lgr5*, *Ascl2* and *Olmf4* [7–9]. More recently a second population of slow cycling/quiescent cells has been noted to reside at the fourth position from the crypt base (+4) [10, 11] and *Bmi1*, *Tert* and *Hopx* amongst other genes have been proposed as markers of this +4 population [12–17]. However the +4 model has been challenged with a number of studies demonstrating the expression of these markers also within the Lgr5+ population of CBCs [15, 18]. A more general model of crypt plasticity attempts to reconcile both of these observations and suggests the existence of two populations of stem cells; one actual and one in reserve that can be called upon in response to tissue damage [16].

The WNT pathway plays a crucial role in intestinal proliferation and ISC maintenance as evidenced by ablation of the major WNT target gene T cell factor 4 (TCF4), which causes a loss of proliferative crypts [19]. A primary point of regulation of the WNT pathway is at the level of the destruction complex. In the absence of WNT proteins, the kinase GSK3 along with β-catenin, Adenomatous Polyposis Coli (APC), Casein Kinase 1 (CK1) and the scaffolding protein AXIN form this complex. In the basal state, GSK3 is active and phosphorylates β-catenin leading to its ubiquitination and subsequent degradation [20, 21]. Upon WNT binding to Frizzled receptors and low-density lipoprotein receptor-related protein (LRP) co-receptors and subsequent engagement of Dishevelled, the destruction complex is antagonised and cytoplasmic β-catenin avoids phosphorylation by GSK3. These events result in β-catenin accumulation, nuclear translocation and the engagement of TCF/LEF (T-cell factor/lymphoid enhancer-binding factor) to activate the expression of downstream genes.

GSK3 has two isoforms, GSK3α and GSK3β, encoded by different genes and these kinases share over 97% sequence homology within their catalytic domains [22]. GSK3α and GSK3β are redundant in the WNT pathway [23] but the mechanisms underpinning WNT-induced inhibition of β-catenin phosphorylation by GSK3 are incompletely understood. Initially, parallels were drawn to the inactivation of GSK3 in the insulin signalling pathway whereby GSK3α/β is inactivated through inhibitory phosphorylation at residues S21/S9 by Protein Kinase B (PKB) [24]. Phosphorylation of GSK3α/β in this manner was shown to cause the N-terminal tail of GSK3 to associate in the substrate-binding pocket, preventing the binding of primed substrates [25]. However, subsequent analysis has shown that GSK3 regulation occurs through distinct mechanisms in the two pathways and that WNT signalling does not lead to a detectable change in S9 phosphorylation of GSK3β [26]. More recent investigations have suggested alternative models to explain how WNTs inhibit GSK3 [27, 28]. One hypothesis is that phosphorylated PPPSPxS motifs in the cytoplasmic tail of LRP act as a competitive pseudosubstrate in the GSK catalytic pocket, thus preventing substrate phosphorylation through GSK3 inhibition [29, 30]. A second proposes that GSK3 and β-catenin are spatially separated by the uptake of GSK3 into multivesicular bodies (MVBs), preventing cytoplasmic β-catenin phosphorylation [31]. Another model posits disruption of the destruction complex through APC/Axin dissociation from GSK3/β-catenin [32, 33].

To investigate GSK3 regulation, and the specific role of N-terminal serine phosphorylation, in a biologically relevant tissue, we have analysed the intestinal phenotype of knockin mice in which serines 21 and 9 of GSK3α and GSK3β respectively have been mutated to alanine [34]. *In vivo*, we find no role for this phosphorylation event in WNT signalling or gut homeostasis although a small effect on intestinal apoptosis is observed. These data confirm that GSK3 regulation in the context of the WNT pathway must occur through mechanisms independent of N-terminal serine phosphorylation.

## Materials and Methods

### Experimental animals

Animal experiments were performed under Home Office project license authority. The GSK3α^S21A/S21A^/β^S9A/S9A^ (GSK3^KI/KI^) mice used for this study have been previously reported [34] and were kindly provided by Dario Alessi, MRC Phosphorylation Unit, Dundee, UK. Mice were maintained on the C57BL6 background and male and female mice were randomly selected for all experiments. For the survival data, animals were kept on study until they became moribund and were sacrificed humanely according to Home Office regulations. For all other experiments, all mice used were 9–10 weeks of age and none of these became ill or died before this time point. For proliferation analysis, mice were injected with 1mg of BrdU by intraperitoneal injection and harvested 3 hours later [35].

### Genotyping of mice

Genotyping of GSK3α^21A^ and GSK3β^9A^ mice was carried out by PCR of genomic DNA isolated from ear samples as described [34]. Primers for GSK3α^21A^ were: P1 (5’-TTGAAGTGGCTGGTACTGGCTCTG-3’) and P2 (5’- GTGTGCTCCAGAGTAGTACCTAGC-3’), resulting in a 271 base pair product for the wild type allele and a 317 base pair product for the mutant allele. Primers for GSK3β^9A^ were: P3 (5’- TCACTGGTCTAGGGGTGGTGGAAG-3’) and P4 (5’-GGAGTCAGTGACAACACTTAACTT-3’), resulting in a 233 base pair product for the wild type allele and a 352 base pair product for the mutant allele.

### Processing of tissue and staining

The small intestine was removed, flushed with PBS and cut into 6 equal segments that were cut longitudinally and “swiss-rolled” as described previously [36]. Each roll was placed in 4% (w/v) paraformaldehyde (PFA) in PBS and rocked gently for 16–18 hr. The PFA was decanted and the tissue was washed and stored in 70% (v/v) ethanol at 4°C. Tissue was processed and embedded in paraffin for sectioning. H&E staining was performed as previously described [35]. PAS staining was carried out according to the manufacturer’s instructions using the PAS Kit (Thermo Scientific, 87007). For AB-PAS staining, sections were de-paraffinized, re-hydrated and Alcian Blue was added for 30 min (1% Alcian blue pH2.5 in 3% glacial acetic acid). Sections were washed for 5 min before continuing with the PAS kit instructions. Alkaline phosphatase staining was carried out using the BCIP/NBT Liquid Substrate System according to the manufacturer’s instructions (Sigma, B1911). Stained sections were analysed with a Leica DFC420 light microscope and photographed using LAS V4.5.

### Immunohistochemistry

5 μm sections of paraffin-embedded tissue were cut using a microtome, de-paraffinized, rehydrated, and endogenous peroxidase activity was quenched with 3% H_2_O_2_ in methanol for 15 min. Tissue sections were microwaved in a pressure cooker in 0.01 M sodium citrate (pH 6.0) to retrieve the antigenicity for 15 min, and then the slides were allowed to cool to room temperature. Sections were washed in PBS before being blocked with 5% swine/rabbit serum in PBS for 1h prior to incubation with primary antibody in 5% serum-containing PBS overnight at 4°C. After washing, biotinylated secondary antibodies (DAKO) were applied for 1h. After subsequent washing, Horseradish Peroxidase Streptavadin conjugate (Vector Laboratories) was applied for 30 min followed by peroxidase detection with DAB (Vector Laboratories) and counterstaining with haematoxylin or eosin. Finally, the sections were dehydrated and mounted with DPX (Sigma, 06522). Primary antibodies used were: BrdU (Cell signalling, 5292, 1:500), β-catenin (BD Transduction, 610153, 1:150), Phospho-histone H3 (Cell signalling, 9701, 1:500), Lysozyme (DAKO, A0099, 1:2000) and Synaptophysin (Abcam, ab52636, 1:200).

### Crypt and villi measurements

Villi and crypt length measurements were taken using the Leica light microscope as above. For crypt cell counts, only well-orientated crypts were counted and a minimum of 50 crypts per animal was scored for each experiment. All counting and measurements were undertaken blind and conducted on at least 3 animals per genotype.

### Western blot analysis

To prepare protein lysates, snap-frozen small intestine samples were homogenized on ice for 1 min in ice-cold RIPA lysis buffer (150 mM NaCl, 50 mM Tris pH 7.0, 5 mM EDTA, 5 mM EGTA, 1% Triton X-100 and 0.5% NP40) supplemented with protease and phosphatase inhibitor cocktail (Calbiochem 539134 and Sigma P0044 respectively). Samples were centrifuged at 14,000 x g for 10 min at 4°C to remove insoluble debris and supernatant collected. The protein concentration of supernatants was determined by the Bradford's method (Pierce). Primary antibodies used were: β-Catenin (BD Transduction 610153), phospho-GSK3α/β (Ser21/9) (Cell Signalling 9331), GSK3α/β (Cell Signalling 5676), ERK2 (Santa Cruz sc-1647) GAPDH (Millipore, MAB374), cleaved Caspase 3 (Cell Signalling 7661) and cleaved PARP (Enzo BML-SA249).

### Expression analysis

RNA extraction and cDNA synthesis were performed using the RNeasy Mini Kit (Qiagen, 74104) and SuperScript III Reverse Transcriptase (Invitrogen 18080–093) kits respectively following the manufacturers' instructions. qRT-PCR was performed using Bioline Sensifast SYBR No-ROX (BIO-98020) on a Roche Light Cycler 480 real-time cycling machine as outlined by the manufacturer and described in [37]. Each sample was analysed in triplicate and three biological replicates were utilised for each experiment. For stem cell marker genes, the expression level was normalized against the housekeeping gene *Aprt1* and the expression values of KI/KI samples relative to that of WT/WT mice were expressed as fold change. Primers used for stem cell marker genes were: *Aprt1* (For: 5’-GTCATTGTGGATGACCTCC-3’ and Rev: 5’-CCACCAAGCAGTTCCTG-3’), *Cyclin D1* (For: 5’-CGGATGAGAACAAGCAGACC-3 and Rev: 5’-TGGAAAGAAAGTGCGTTGTG-3’), *Axin2* (For: 5’-ATGAGTAGCGCCGTGTTAGTG-3’ and Rev: 5’-GGGCATAGGTTTGGTGGACT-3’), *Lgr5* (For: 5’-CCCAATGCGTTTTCTACGTT-3’ and Rev: 5’-TAACCCAGTCACAGGGAAGG-3’), *Ascl2* (For: 5’- GGTGACTCCTGGTGGACCTA-3’ and Rev: 5’- TCCGGAAGATGGAAGATGTC-3’), *C-myc* (For: 5’-TCTCCACTCACCAGCACAACTACG-3’ and Rev: 5’-ATCTGCTTCAGGACCCT-3’), *Bmi1* (For: 5’- GAGCAGATTGGATCGGAAAG-3’ and Rev: 5’-GCATCACAGTCATTGCTGCT-3’), *Hopx* (For: 5’- CAACTTCAACAAGGTCAACAAGC-3' and Rev: 5’-GCTTAAACCATTTCTGCGTC-3’), *Tert* (For: 5’- CAGCCATACATGGGCCAGTTC-3’ and Rev: ACAGGCTGCTGCTGCTCTCA-3’). For analysis of the Notch pathway, ready-made individual primers encoding regulatory genes of differentiation were used (QuantiTect Primer Assay, Qiagen). The mRNA expression level of these genes was standardized against the geometric mean of three housekeeping genes: *Aprt1*, *Hprt1* and *Gusb*.

### RT2 Profiler PCR Array

The Mouse WNT Signalling Targets (PAMM-243Z SABiosciences) was utilised to investigate a panel of 84 WNT specific genes in mouse intestinal tissue as per manufacturer’s instructions. Briefly, 500 ng of RNA was converted to cDNA using RT^2^ First Strand Kit (SABiosciences). The resultant cDNA product was immediately amplified by qPCR using RT^2^ SYBR Green qPCR Master Mix (SABiosciences) and a Roche Lightcycler 480. The Ct values (threshold cycle) for both KI/KI and WT/WT samples were evaluated using the provided web-based portal (http://pcrdataanalysis.sabiosciences.com/pcr/arrayanalysis.php) and normalised to housekeeping genes present on the array. This analysis generated fold-change, which represents the normalized gene expression in the KI/KI samples compared to the normalized gene expression in the WT/WT samples. Following the portal guidelines, a significant difference in expression was set at greater than or less than 3-fold difference in fold-change.

### Statistical analysis

Comparison between any two groups was performed by an unpaired t-test assuming Gaussian distribution.

## Results

### Phenotype of GSK3α/β^KI/KI^ mutant mice

To obtain GSK3α/β single and double knockin mutant mice, GSK3α^+/S21A^ animals were first intercrossed with GSK3β^+/S9A^ animals. GSK3α^+/S21A^;GSK3β^+/S9A^ double heterozygous mice in the offspring were then subjected to a second round of breeding by intercrossing (Mating 1 in Table 1). An additional round of breeding was performed to generate further double homozygous animals by intercrossing male and female GSK3α^S21A/S21A^;GSK3β^S9A/S9A^ mice (Mating 2 in Table 1). Consistent with the results of McManus et al [34], double homozygous GSK3α^S21A/S21A^;GSK3β^S9A/S9A^ mice were born alive in both matings and survived to adulthood.

**Table 1:** Genotypes of mice reported in this study.

Genotype of mouse	Observed number (%)	Expected number (%)
**Mating 1**^a^: GSK3α^+/21A^; GSK3β^+/9A^; X; GSK3α^+/21A^; GSK3β^+/9A^		
Genotypes of offspring from 18 litters with 6.6 average litter size		
GSK3α^+/+^; GSK3β^+/+^	18 (15%)	7.5 (6.25%)
GSK3α^+/+^; GSK3β^+/9A^	13 (10.7%)	15 (12.5%)
GSK3α^+/+^; GSK3β^9A/9A^	11 (9.2%)	7.5 (6.25%)
GSK3α^+/21A^; GSK3β^+/+^	18 (15%)	15 (12.5%)
GSK3α^+/21A^; GSK3β^+/9A^	23 (19.2%)	30 (25%)
GSK3α^+/21A^; GSK3β^9A/9A^	11 (9.2%)	15 (12.5%)
GSK3α^21A/21A^; GSK3β^+/+^	8 (6.7%)	7.5 (6.25%)
GSK3α^21A/21A^; GSK3β^+/9A^	14 (11.7%)	15 (12.5%)
GSK3α^21A/21A^; GSK3β^9A/9A^	4 (3.3%)	7.5 (6.25%)
**Total**	**120 (100%)**	**120 (100%)**
**Mating 2**^b^: GSK3α^21A/21A^; GSK3β^9A/9A^ X GSK3α^21A/21A^; GSK3β^9A/9A^		
Genotype of offspring from 6 litters with 3.8 average litter size.		
GSK3α^21A/21A^; GSK3β^9A/9A^	23 (100%)	23 (100%)

^a^For Mating 1, double heterozygous GSK3α^+/21A^; GSK3β^+/9A^ male and female mice were intercrossed with each other. Offspring, post 6 weeks of age, were genotyped and the expected and observed numbers of mice of each genotype are shown.

^b^For Mating 2, double homozygous GSK3α^21A/21A^; GSK3β^9A/9A^ mice were intercrossed and surviving offspring, post 6 weeks of age, were genotyped. As expected, 100% of mice had the GSK3α^21A/21A^; GSK3β^9A/9A^ genotype.

**Fig 1:**
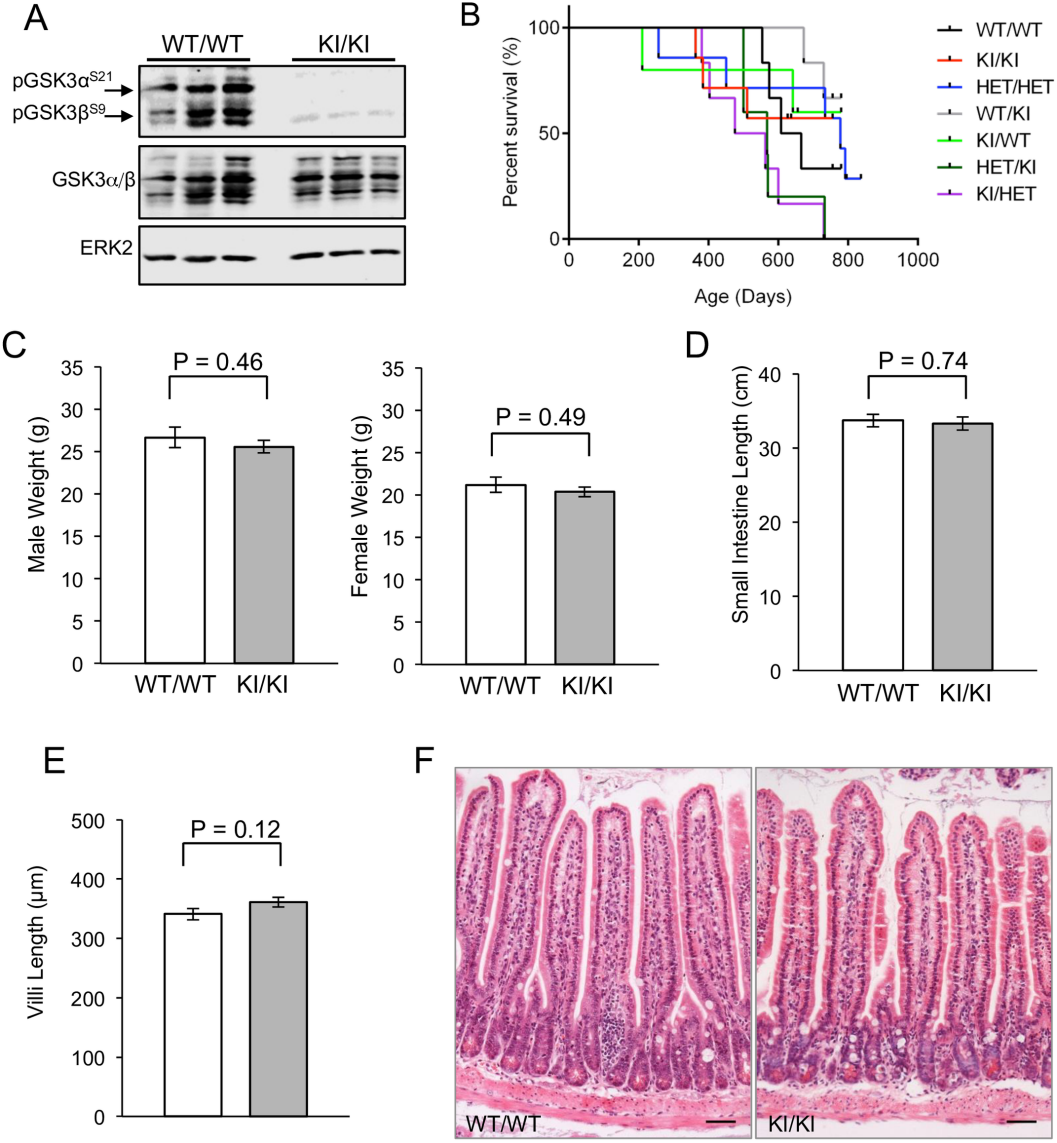
GSK3α/β^KI/KI^ phenotype. A: Immunoblots show no phosphorylation of serine 21 of GSK3α or serine 9 of GSK3β in the GSK3α/β^KI/KI^ animals. Protein lysates derived from the small intestine of control wild-type or mutant GSK3α/β^KI/KI^ mice were analysed with an antibody for phosphorylated GSK3α/β. Immunoblotting for GSK3α/β and ERK2 was used to determine equal protein loading. B: Kaplan-Meier survival analysis of GSK3α/β mutant mice. All mice were kept on study until they became moribund. None of these animals demonstrated specific symptoms prior to this. Genotypes of mice are as follows: WT/WT = GSK3α^+/+^;GSK3β^+/+^ (n = 6), KI/KI = GSK3α^S21A/S21A^;GSK3β^S9A/S9A^ (n = 7), HET/HET = GSK3α^+/S21A^;GSK3β^+/S9A^ (n = 7), WT/KI = GSK3α^+/+^;GSK3β^S9A/S9A^ (n = 6), KI/WT = GSK3α^S21A/S21A^;GSK3β^+/+^ (n = 5), HET/KI = GSK3α^+/S21A^;GSK3β^S9A/S9A^ (n = 5), KI/HET = GSK3α^S21A/S21A^;GSK3β^+/S9A^ (n = 6). Black tick marks show censored data. Median survival was as follows: WT/WT = 637d, KI/KI = 686d, HET/HET = 778d, WT/KI = undefined, KI/WT = undefined, HET/KI = 568d and KI/HET = 519d. C: Weight analysis. Age matched males and females were weighed at 10 weeks of age. Genotypes of mice were: WT/WT = GSK3α^+/+^;GSK3β^+/+^ (n = 3), KI/KI = GSK3α^S21A/S21A^;GSK3β^S9A/S9A^ (n = 3) for each sex. Mean + SEM are shown, demonstrating no statistically significant difference in the mean weights for any group using the Student’s t-test. D: Intestine length. The small intestine length was measured and mean length + SEM is shown. Genotypes of mice were: WT/WT = GSK3α^+/+^;GSK3β^+/+^ (n = 6), KI/KI = GSK3α^S21A/S21A^;GSK3β^S9A/S9A^ (n = 7). No statistically significant difference was observed using the Student’s t-test. E: Villus length. The length of villi was measured in age matched males and females at 10 weeks of age. Genotypes of mice were: WT/WT = GSK3α^+/+^;GSK3β^+/+^ (n = 5), KI/KI = GSK3α^S21A/S21A^;GSK3β^S9A/S9A^ (n = 7). 50 villi were measured per animal. No statistically significant difference was observed using the Student’s t-test. F: H&E stained small intestinal sections of GSK3α/β^WT/WT^ or mutant GSK3α/β^KI/KI^ mice are shown. Scale bars, 50μm.

Protein lysates were generated from the intestine of GSK3α^S21A/S21A^;GSK3β^S9A/S9A^ (called KI/KI) animals and immunoblot analysis with antibodies for phosphorylated forms of S21 of GSK3α and S9 of GSK3β confirmed inheritance of mutated knockin alleles (Fig 1A). Consistent with previous reports [34], the single and double homozygous and heterozygote knockin animals demonstrated no significant difference in survival compared to controls (Fig 1B) and appeared overtly normal. Male and female weights at 10 weeks were indistinguishable from that of wild-type controls (Fig 1C). A decrease in villus number and loss of the proliferative compartment has been previously reported in mice with defective WNT signalling [19, 38]. However the length of the small intestine was not significantly different in the GSK3 knockin mice compared to wild type controls (Fig 1D) and neither was the length of the villi (Fig 1E). Furthermore, Haematoxylin & Eosin (H&E) staining of small intestine sections demonstrated normal tissue architecture and integrity (Fig 1F).

### Intestinal cell proliferation and apoptosis

**Fig 2:**
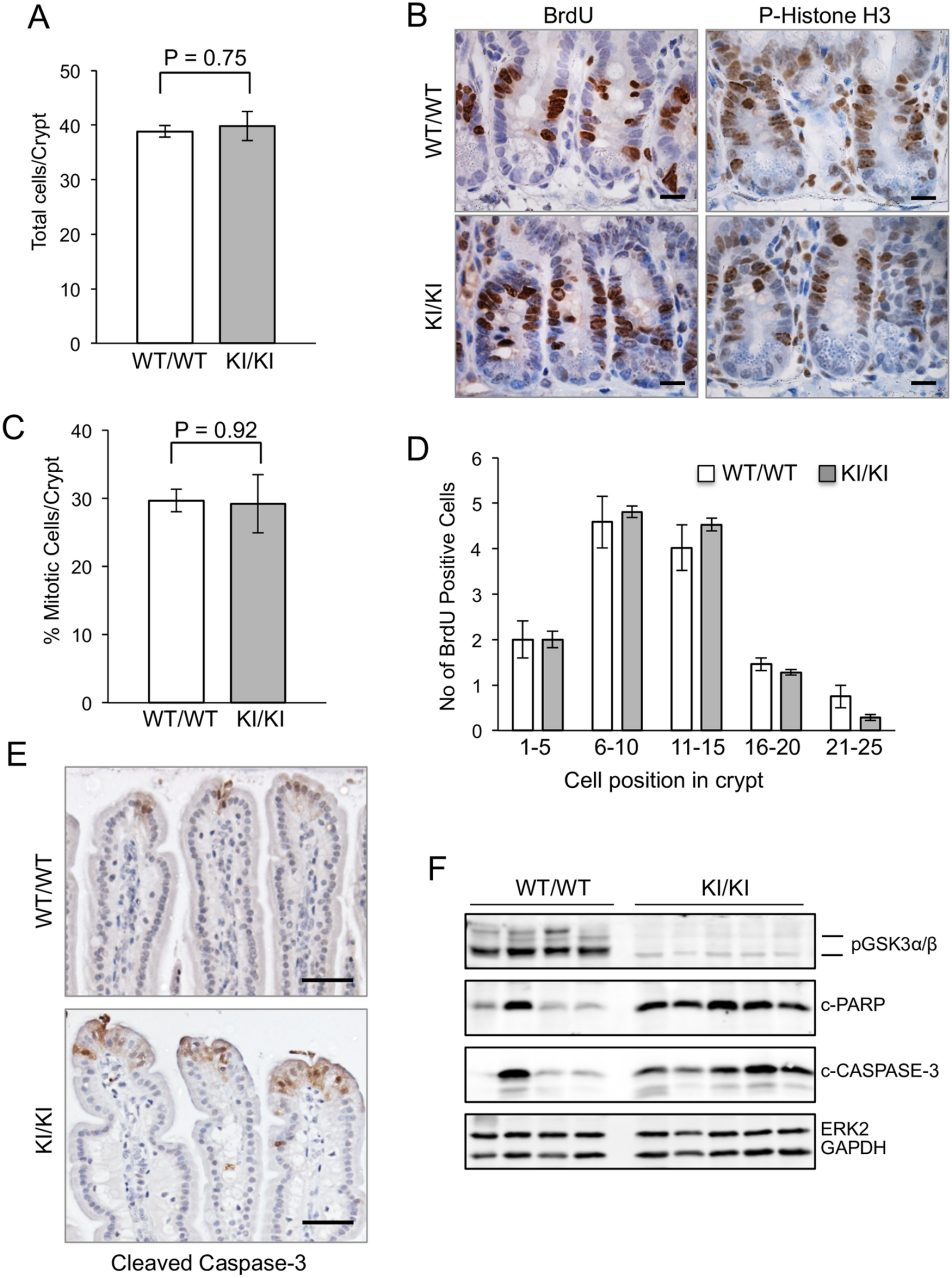
Proliferation and apoptosis analysis of the small intestine. A: Quantification of the total number of cells per crypt. A minimum of 50 crypts were quantitated per animal of each genotype and mean values are shown + SEM. Genotypes were: GSK3α^+/+^;GSK3β^+/+^ (n = 3), KI/KI = GSK3α^S21A/S21A^;GSK3β^S9A/S9A^ (n = 3). No statistically significant difference was observed using the Student’s t-test. B: Immunohistochemical staining of WT/WT and KI/KI small intestinal crypts using antibodies for BrdU or phospho-Histone H3. Scale bars, 20μm. C: Quantification of BrdU-positive cells per crypt within the WT/WT and KI/KI small intestine. Staining in 50 crypts of three mice of each genotype was quantified. No statistically significant difference was observed using the Student’s t-test. D: The position of BrdU-positive cells within the WT/WT and KI/KI crypts was quantified. Position 0 represents the base of the crypt. A minimum of 50 crypts were quantified in three mice of each genotype. No statistically significant difference was observed using the Student’s t-test. E: Immunohistochemical staining of WT/WT and KI/KI villi using an antibody for cleaved caspase 3. Scale bars, 50μm. F: Western blot analysis of small intestinal protein samples from WT/WT or KI/KI mice using antibodies for phosphorylated GSK3α/β, cleaved PARP or cleaved caspase-3. Immunoblotting for ERK2 and GAPDH was used to determine equal protein loading.

To further assess whether the GSK3 mutations affect intestinal homeostasis, we assessed proliferation and apoptosis in the crypts and villi. Total numbers of cells per intestinal crypt were not significantly different in the KI/KI animals compared to wild-type controls (Fig 2A). To investigate proliferation in more detail, BrdU incorporation was assessed, but demonstrated no difference between the knockin animals and controls (Fig 2B and 2C). Immunohistochemical staining for phosphohistone-H3 also demonstrated no difference in staining pattern (Fig 2B). In both KI/KI and WT/WT mice, cycling cells were confined to the mid crypt region with there being no difference in the localisation of these cells in the mutant mice (Fig 2D).

To assess apoptosis, immunostaining was performed using an antibody for cleaved caspase 3 and this demonstrated a moderate increase in staining in the villi tips of the KI/KI animals compared to controls (Fig 2E). An increase in levels of apoptosis was supported by immunoblotting, demonstrating increased levels of cleaved PARP and cleaved caspase 3 in the majority of the KI/KI intestinal samples compared to controls (Fig 2F).

### Intestinal cell differentiation

**Fig 3:**
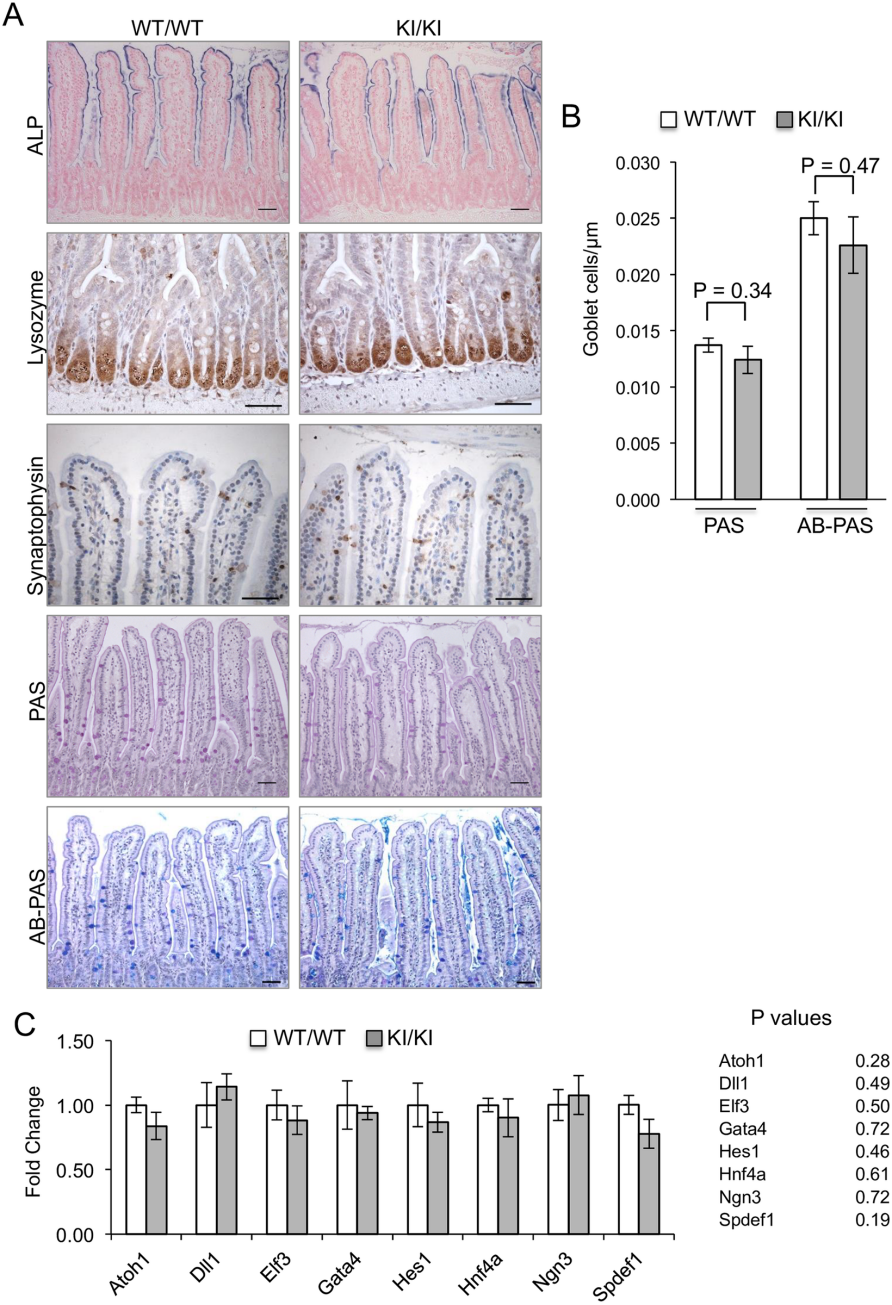
Cell differentiation analysis. A: Histological sections of the small intestine from WT/WT or KI/KI mice were analysed by immunostaining with antibodies for ALP (differentiated enterocytes), Lysozyme (Paneth cells) or Synaptophysin (enteroendocrine cells). Histological sections were counterstained with eosin in the case of the ALP staining and haematoxylin in the case of Lysozyme and Synaptophysin staining. In the bottom two panels, sections were subjected to PAS and AB-PAS staining to detect neutral and acid mucin-producing goblet cells respectively. Scale bars, 50μm. B: Quantification of goblet cell number. PAS+ and AB-PAS+ cells were quantified in the villi of WT/WT and KI/KI mice. A minimum of 50 villi were quantitated per animal from at least five mice of each genotype and mean values are shown + SEM. Cell counts were adjusted according to villi length. No statistically significant difference was observed using the Student’s t-test. C: Expression of genes involved in intestinal differentiation. Real-time PCR analysis was used to determine transcript levels in RNA specimens of the small intestine derived from three WT/WT or three KI/KI mice for a panel of 8 genes involved in epithelial differentiation. Shown are the fold-changes in transcript levels between KI/KI and WT/WT mice. No statistically significant difference in the expression of any of the eight genes was observed using the Student’s t-test (P values for each gene are shown).

We next examined if the GSK3 mutant mice had altered patterns of intestinal cell differentiation. Differentiated enterocytes were stained with Alkaline Phosphatase (ALP), Paneth cells with Lysozyme and enteroendocrine cells with Synaptophysin (Fig 3A). The level of staining and location of these cells was not noticeably altered between the KI/KI animals and controls. Periodic acid-Schiff (PAS) and Alcian Blue (AB)-PAS stains demonstrated normal distribution of goblet cells producing neutral and acid mucins (Fig 3A). The number of goblet cells, as determined by quantitating (PAS) and (AB-PAS) staining, was also not significantly different between KI/KI animals and controls (Fig 3B).

To further confirm that patterns of cell differentiation are not affected by the GSK3α/β knockin mutations, we undertook quantitative gene expression analysis of key genes known to be involved in intestinal epithelial cell differentiation namely *Atoh1*, *Dll1*, *Elf3*, *Gata4*, *Hes1*, *Hnf4a*, *Ngn3* and *Spdef1*. The expression of these genes was not significantly different between KI/KI intestinal samples and controls (Fig 3C).

### WNT pathway activation and stem cell homeostasis

**Fig 4:**
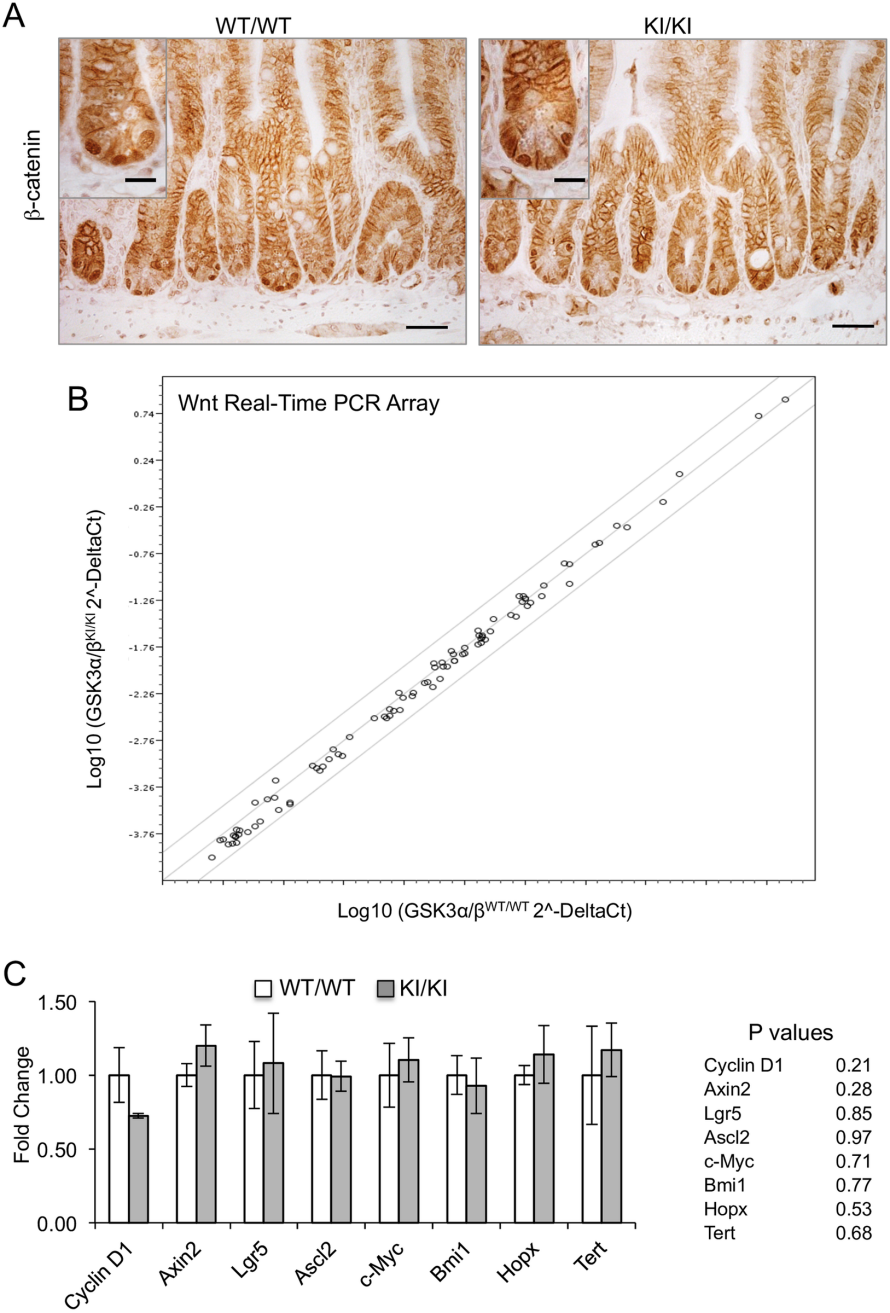
Analysis of the WNT signalling pathway. A: Immunohistochemical staining of β-catenin in the small intestine of WT/WT and KI/KI mice. Scale bars, 50μm. The inset images show magnified crypts for which the scale bars = 20μm. B: Expression of WNT target genes. Real-time PCR analysis was used to determine transcript levels in RNA specimens of the small intestine derived from three WT/WT or three KI/KI mice for a panel of 84 WNT target genes. The Scatter plot shows the differential expression patterns for each gene between the WT/WT and KI/KI samples (>3 fold up-regulated genes are shown in red, <3 fold down-regulated genes in green, genes showing less than 3 fold up- or down-regulation are shown in black). The scatter plot shows very little deviation of the KI/KI data from the WT/WT trendline. C: Expression of stem cell related genes. Real-time PCR analysis was used to determine transcript levels in RNA specimens of the small intestine derived from three WT/WT or three KI/KI mice for 8 markers of intestinal stem cells. Shown are the fold-changes in transcript levels between KI/KI and WT/WT samples. No statistically significant difference in the expression of any of the eight genes was observed using the Student’s t-test (P values for each gene are shown).

GSK3 is a key point of control of the canonical WNT signalling pathway, with the output of this pathway involving nuclear β-catenin translocation and transactivation of WNT target genes. To address the involvement of N-terminal serine phosphorylation of GSK3 in the canonical WNT signalling pathway in the intestine, immunohistochemical staining for β-catenin was performed using KI/KI samples and controls (Fig 4A). β-catenin localisation was observed in crypt base cells in tissue derived from both sets of mice and there was no difference in the number or pattern of nuclear β-catenin staining (Fig 4A). To further examine the consequence of GSK3 mutation on the WNT pathway we assessed the expression of 84 WNT target genes using a real-time PCR array. The difference in expression of these genes in the KI/KI intestine compared to control intestine is shown in the scatter plot in Fig 4B. The data show no significant difference in the expression of any of these genes.

The WNT signalling pathway is known to play a key role in stem cell maintenance and fate in the mouse small intestine and ISCs have been identified at the +4 position as well as in the crypt base [39]. The +4 stem cells and CBCs have been distinguished based on expression of key genes with *Lgr5* and *Ascl2* identifying CBCs and *Bmi1*, *Tert* and *Hopx* thought to distinguish +4 cells [39]. To investigate if GSK3 phosphorylation plays a role in stem cell maintenance, the expression of these genes and other genes known to be responsible for the proliferative effects of the WNT pathway namely *Axin2*, *Ccnd1* and *c-Myc* [40, 41] were examined using quantitative RT-PCR. We found no significant difference in the expression of these genes in the KI/KI intestine compared to controls (Fig 4C).

## Discussion

GSK3 is a conserved serine-threonine kinase that was originally identified as an important regulator of insulin-dependent glycogen synthesis [42]. GSK3, and specifically its two isoforms GSK3α and GSK3β, has since been shown to have a wide range of substrates and be involved in multiple physiological processes including apoptosis, cell differentiation and proliferation. As a consequence GSK3 has been implicated in many human pathologies including cancer, diabetes and Alzheimer’s disease [28, 43]. Given its pleiotropic functions and therapeutic potential, the mode of regulation of GSK3 under different cellular contexts and conditions has been the subject of intensive investigation.

GSK3 kinase activity is inhibited through phosphorylation of serine residues within its N-terminal region and, specifically, serine 21 in GSK3α and serine 9 in GSK3β. A number of kinases have been shown to phosphorylate these residues including PKB/AKT, p70^S6K^ and p90^RSK,^ with PKB/AKT targeting GSK3 phosphorylation in response to insulin signalling. Within the WNT signalling pathway, GSK3 is also subject to regulation and phosphorylation of serines 21/9 has been suggested to play a role in this [44, 45]. However, this mode of regulation has been contradicted by more recent observations [26, 43], including the demonstration that embryonic stem (ES) cells carrying homozygous serine-alanine mutations at residues 21/9 of GSK3 α/β do not have disrupted WNT signalling [34]. In this manuscript we have extended the analysis to a more physiologically relevant setting, and specifically through analysis of the intestine, which is known to be dependent on the WNT pathway for renewal and tissue homeostasis [19]. Our data show that, like ES cells, the activity of the WNT pathway is not dependent on N-terminal serine phosphorylation of GSK3 and that these phosphorylation events play only a minor role in maintenance of intestinal integrity. Thus, alternative mechanisms must underpin GSK3 regulation in canonical WNT signalling [27–33].

Homozygous knockin mice with the S21/9A GSK3 mutations survive the normal lifespan of a mouse (Fig 1), are fertile with very little alteration in the anatomical and molecular characteristics of the tissue upon which we have focussed—the small intestine (Figs 1, 2, 3 and 4). However, a small increase in apoptosis levels in the villi tips was observed using immunohistochemical analysis (Fig 2E) and this was validated using western blot analysis (Fig 2F). In line with these data, GSK3β has previously been identified as an anti-apoptotic kinase as evidenced by analysis of *Gsk3β* knockout mice that undergo embryonic lethality due to failure to activate the TNF α/NF-κB survival pathway [46]. Conversely, GSK3 has also been shown to induce apoptosis in response to a number of stimuli including DNA damage, hypoxia and heat shock with multiple mechanisms being reported including modulation of TP53, eIF2B and BCL-2 family members [43, 47]. The opposing roles of GSK3 in apoptosis regulation are likely related to its intricate mode of control and the fact that it has pleiotropic functions and multiple substrates. The specific role of N-terminal GSK3 serine phosphorylation in suppressing or promoting apoptosis has not previously been investigated although a recent *in vitro* study suggests a role of S9 GSK3β phosphorylation in antagonising apoptosis in a NF-κB-dependent manner [48]. Whether this is the case for the intestine requires further experimental investigation.

There is currently considerable interest in targeting GSK3 for therapeutic benefit in cancer. However, this is complicated by the fact that GSK3 has both pro- and anti-tumourigenic functions [28, 47]. Examination of the specific mechanisms by which GSK3 functions in different cancers may allow therapies to be tailored for optimal benefit. To this end, N-terminal serine phosphorylation of GSK3 occurs in response to growth factor signals and GSK3 regulation by this mechanism has been observed in cells bearing oncogenic mutations in growth factor pathway components including PIK3CA, RAS and RAF [35, 49–51]. GSK3 has been shown to mediate oncogenic effects in these contexts including through the stabilisation of Cyclin D1 [49]. Therefore, strategies that reverse N-terminal GSK3 serine phosphorylation may be effective in cancers with the relevant oncogenic mutations. The fact that the S21/9A GSK3α/β homozygous knockin mice survive normally with relatively few pathologies, as shown in this report, is encouraging that any therapy targeting this phosphorylation event would have little effect on normal cells or generate adverse reactions at the tissue or whole organism level.
